# Transfemoral Aortic Valve Implantation with the New Edwards Sapien 3 Valve for Treatment of Severe Aortic Stenosis—Impact of Valve Size in a Single Center Experience

**DOI:** 10.1371/journal.pone.0151247

**Published:** 2016-03-22

**Authors:** Jochen Wöhrle, Birgid Gonska, Christoph Rodewald, Julia Seeger, Dominik Scharnbeck, Wolfgang Rottbauer

**Affiliations:** Department of Internal Medicine II, University of Ulm, Ulm, Germany; Istituto Clinico S. Ambrogio, ITALY

## Abstract

**Aims:**

The third generation Edwards Sapien 3 (Edwards Lifesciences Inc., Irvine, California) system was optimized to reduce residual aortic regurgitation and vascular complications.

**Methods and Results:**

235 patients with severe symptomatic aortic stenosis were prospectively enrolled. Transcatheter aortic valve implantations (TAVI) were performed without general anesthesia by transfemoral approach. Patients were followed for 30 days. Patients received 23mm (N = 77), 26mm (N = 91) or 29mm (N = 67) valve based on pre-procedural 256 multislice computer tomography. Mean oversizing did not differ between the 3 valves. There was no residual moderate or severe aortic regurgitation. Rate of mild aortic regurgitation and regurgitation index did not differ between groups. There was no switch to general anesthesia or conversion to surgery. Rate of major vascular complication was 3.0% with no difference between valve and delivery sheath sizes. Within 30 days rates of all cause mortality (2.6%) and stroke (2.1%) were low.

**Conclusions:**

In patients with severe aortic stenosis transfemoral TAVI with the Edwards Sapien 3 valve without general anesthesia was associated with a high rate of device success, no moderate or severe residual aortic regurgitation, low rates of major vascular complication, mortality and stroke within 30 days with no difference between the 3 valve sizes.

**Trial Registration:**

ClinicalTrials.gov NCT02162069

## Introduction

Transfemoral aortic valve implantation (TAVI) for treatment of symptomatic patients with severe aortic stenosis was associated with a lower long-term mortality compared with patients undergoing surgical valve replacement [1]. Residual paravalvular aortic regurgitation after TAVI has been identified as a significant independent predictor for mortality [2]. In the randomized Placement of Aortic Transcatheter Valves (PARTNER) trial even a mild residual aortic regurgitation (AR) was associated with an increased mortality using the balloon-expandable Edwards Sapien valve [3]. In addition, the occurrence of major vascular bleeding events also independently predict long-term mortality after TAVI [4]. The third generation Edwards Sapien 3 valve (ES3; Edwards Lifesciences, Irvine, CA) has been designed to optimize post-procedural results reducing AR by an outer skirt [5]. In addition, the profile of the delivery system was reduced to 14 French (23 or 26mm valve size) or 16 French (29mm valve size) expandable sheaths in order to reduce vascular and bleeding complications.

We evaluated post-procedural results including residual aortic regurgitation, device success and outcome within 30 days according to the second valve academic research consortium criteria [6] for the three different ES3 valve sizes in a large patient population with severe symptomatic aortic stenosis undergoing transfemoral TAVI without general anesthesia.

## Methods

**Fig 1:**
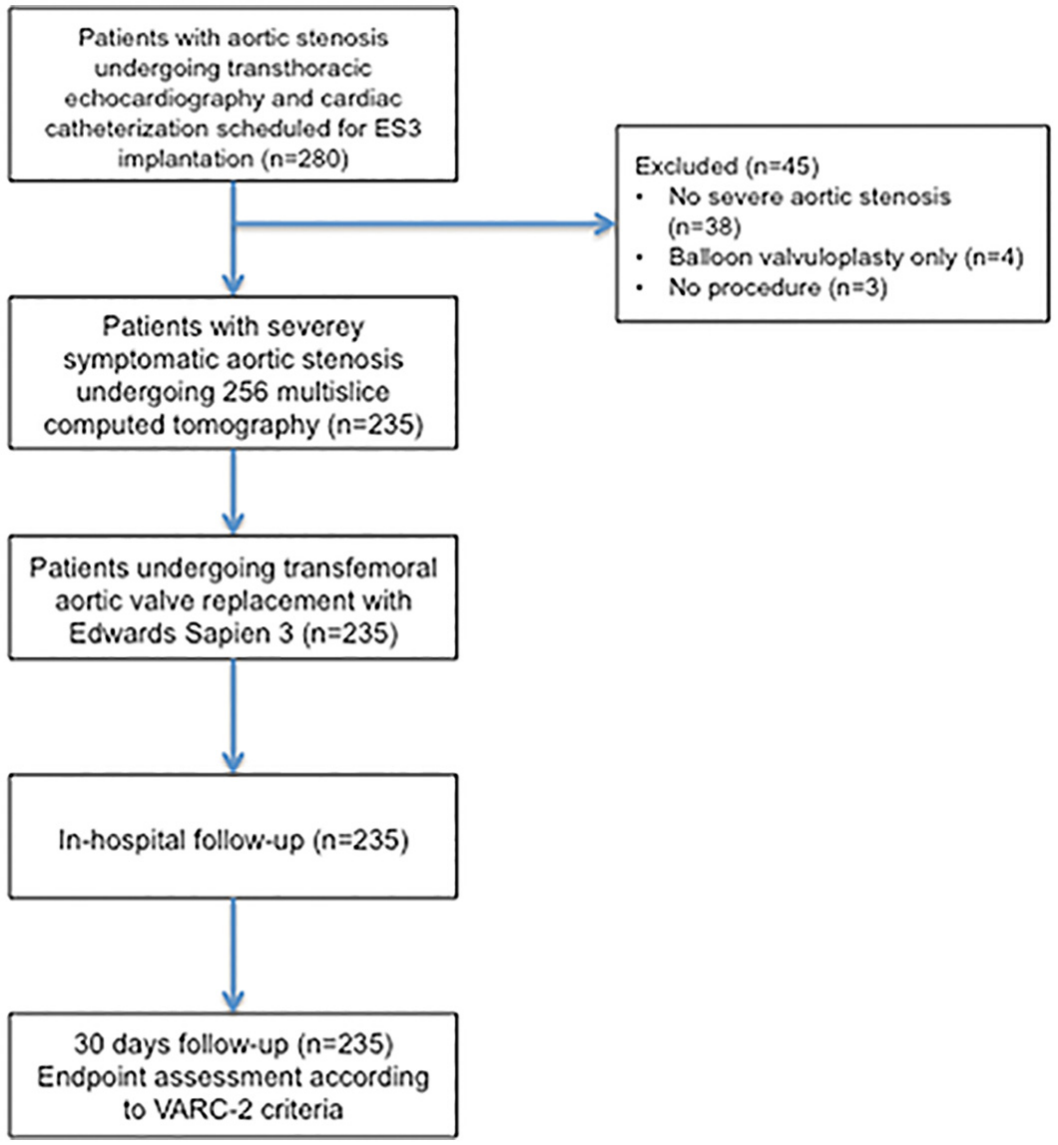
CONSORT flowchart.

**Fig 2:**
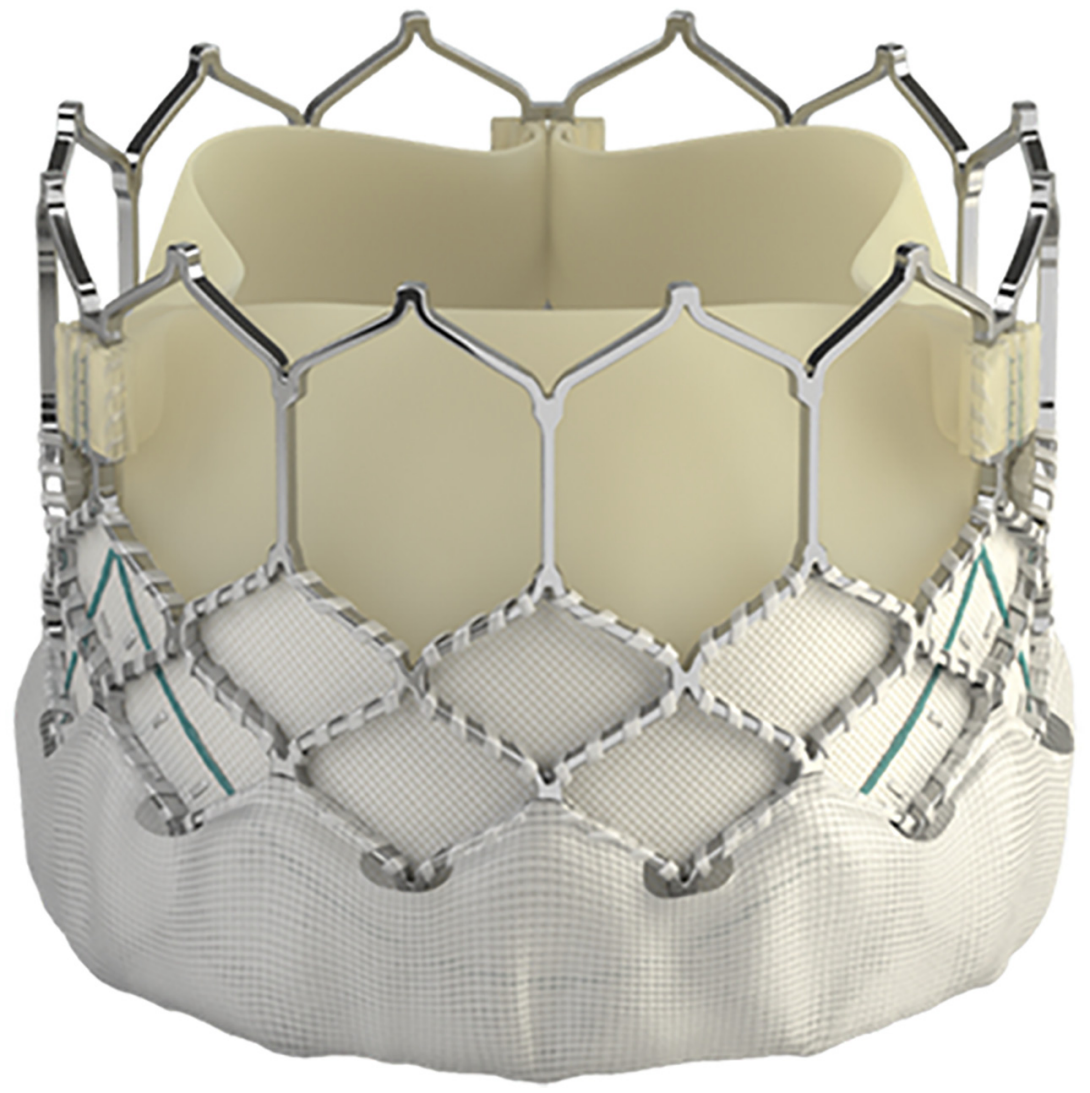
New generation Edwards Sapien 3 valve with the outer skirt (polyethylene terephthalate) to reduce paravalvular aortic regurgitation.

We prospectively evaluated the safety and efficacy of the ES3 valve in 235 patients with severe symptomatic aortic stenosis (Fig 1) and evaluated the impact of valve size. Valve implantation was performed in a hybrid catheterization lab without general anesthesia by transfemoral approach as described elsewhere [7–9]. Patients treated with ES3 (Fig 2) between January 2014 and June 2015 were included and followed for 30 days. All patients suffered from symptomatic severe aortic stenosis documented by echocardiography and cardiac catheterization with an aortic valve area (AVA) ≤ 1cm^2^ or an indexed AVA ≤ 0.6 cm^2^/m^2^. Patients were at intermediate to high risk for surgical valve replacement based on a Society of Thoracic Surgeons (STS) Score for mortality or had relevant comorbidities with contraindications to surgical valve replacement e.g. porcelain aorta, frailty or history of chest radiation. Presence of pulmonary disease was defined as chronic lung disease plus FEV1 <75% of predicted value and chronic inhaled or oral bronchodilator therapy. The heart team including cardiologists and heart surgeons made decision for TAVI. In the study period a total of 362 patients were treated with TAVI. Patients were treated with the ES3 or the Lotus valve (Boston Scientific). Written informed consent was obtained from each patient. The study was ethically approved. The authors confirm that all ongoing and related trials for this intervention are registered (clinicaltrials.gov NCT02162069; S1 CONSORT Checklist, S1 and S2 Protocols).

A pre-procedural 256 multislice computed tomography (Philips Brilliance i-CT256, Philips, Amsterdam, The Netherlands) was used for sizing in all patients. Measurements of aortic annulus, left ventricular outflow tract (LVOT), distance from annulus to coronary ostia, area at the sinotubular junction and area at ostia of the coronary arteries were obtained by a dedicated software (3mensio, 3mensio 7.0 software, Pie Medical Imaging, Maastricht, The Netherlands) in accordance with the guidelines of the Society of Cardiovascular Computed Tomography [10]. Aortic cusp calcification was assessed according to Rosenhek [11]. Oversizing or undersizing was calculated as % oversizing = (ES3 nominal area/annular area by computer tomography—1)*100. Nominal areas for 23, 26 or 29mm ES3 valve were 406, 519 and 649mm^2^ as described elsewhere [12].

The ES3 valve was implanted with fluoroscopic guidance and rapid pacing in the orthogonal view of the annulus. Aortic regurgitation after TAVI was analyzed by standardized aortography [7,13]. Aortic regurgitation index was calculated as described elsewhere [14]. Aortic regurgitation and measurement of pressure gradients by transthoracic echocardiography was done one day after valve implantation. Aortic regurgitation was graded none, trace, mild, moderate or severe as described elsewhere [1,7,15].

Post-procedural outcome was analyzed according to the Valve Academic Research Consortium-2 (VARC-2) criteria [6] evaluating post-procedural aortic regurgitation and device success. Device success was defined as the absence of procedural mortality and correct positioning of a single prosthetic heart valve into the proper anatomical position and intended performance of the prosthetic heart valve (no prosthesis-patient mismatch and mean aortic valve gradient <20 mmHg or peak velocity <3 m/sec, and no moderate or severe prosthetic valve regurgitation). Patients were followed for 30 days to assess the early safety endpoint according to the VARC-2 criteria.

### Statistical analysis

For statistical analyses the Statistica software version 10 (Stat Soft, Inc, Tulsa, OK, USA) was used. Pre-procedural data and post-procedural results were compared between the 3 different valve sizes (23, 26 and 29mm). Continuous variables are expressed as mean±one standard deviation and were compared with ANOVA testing. Normal distribution of continuous variables was tested using the Kolmogorov-Smirnov test. Categorical variables are presented as counts and percentages and differences between proportions were calculated by using Chi^2^ test. A value of p <0.05 was considered statistically significant.

## Results

Patients had multiple comorbidities (Table 1) with a predicted risk of operative mortality of 7.0±5.0 by STS and by 18±14 by logistic EuroScore estimates. Forty-one patients (17.4%) had a STS score for mortality <4%, 121 patients (51.5%) a STS score 4–8% and 73 patients (31.1%) a STS score >8%. History of cardiac surgery was present in 13%, presence of pulmonary disease in 60% and atrial fibrillation in 43% of patients. Patients suffered from single vessel coronary artery disease in 16% (N = 38), two vessel disease in 17% (N = 40) and three-vessel disease in 28% (N = 65). American Society of Anesthesiologists score for perioperative risk was 3.5±0.6. Transthoracic echocardiography and cardiac catheterization demonstrated severe aortic stenosis (Table 2). The majority of patients (86%) were severely symptomatic with NYHA class III or IV. Detailed analyses of the multislice computed tomography for the 3 valve sizes are shown in Table 3. Calcifications of aortic cusps or LVOT were similar between the 3 groups. Measurements of the annulus and LVOT parameters were significantly different for the 23, 26 and 29mm valve sizes with a perimeter derived diameter of the aortic annulus of mean 22.8mm for the 23mm group (N = 77), 25.7mm for the 26mm group (N = 91) and 28.4mm for the 29mm population (N = 67). Distance from annulus to left or right coronary ostium was shortest in the 23mm population and longest in the 29mm group. There was no difference in rate of oversizing between the 3 valve sizes ranging between mean 4.8 and 8.1%.

**Table 1:** Baseline clinical characteristics.

	Edwards Sapien 3
Number of patients, N	235
Age, years (range)	80.7 ± 6.2 (61–100)
Female	120 (51.1%)
BMI (kg/m^2^)	27.1±4.8
Diabetes mellitus	76 (32.3%)
Severe chronic renal failure	101 (43.0%)
Coronary artery disease	143 (60.9%)
History of myocardial infarction	42 (17.9%)
History of cardiac surgery	31 (13.2%)
Peripheral or cerebral vascular disease	27 (11.5%)
History of stroke or intracerebral bleeding	34 (14.5%)
Pulmonary disease	140 (59.6%)
Atrial fibrillation	102 (43.2%)
Permanent pacemaker	27 (11.5%)
Logistic EuroScore	17.6 ± 14.3
STS for mortality	7.0 ± 5.0

Values are mean ± SD or n (%); BMI = Body mass index

**Table 2:** Baseline aortic valve parameters.

	Edwards Sapien 3
**Transthoracic echocardiography**	
Aortic valve area, cm^2^	0.77±0.22
Indexed aortic valve area, cm^2^/m^2^	0.28±0.11
Mean gradient, mmHg	35.2±14.3
Maximum gradient, mmHg	61.8±22.4
Moderate/severe aortic regurgitation	32 (13.6%)
**Cardiac catheterization**	
Aortic valve area, cm^2^	0.67±0.23
Indexed aortic valve area, cm^2^/m^2^	0.24±0.08

Values are mean ± SD or n (%).

**Table 3:** Baseline computer tomographic parameters.

	All patients	23mm	26mm	29mmm	P value
Number of patients, N	235	77	91	67	
**Aortic annulus diameter, mm**					
Area derived diameter	25.0 ± 2.5	22.2 ± 1.3	25.1 ± 1.6	27.7 ± 1.0	<0.001
Perimeter derived diameter	25.6 ± 2.6	22.8 ± 1.4	25.7 ± 1.7	28.4 ± 1.0	<0.001
Area, mm^2^	494 ± 101	387 ± 45	495 ± 69	604 ± 44	<0.001
Perimeter	80.4 ± 8.1	71.6 ± 4.4	80.9 ± 5.4	89.1 ± 3.1	<0.001
Maximum diameter	28.0 ± 3.0	25.0 ± 2.1	28.0 ± 2.1	31.1 ± 1.3	<0.001
Minimal diameter	22.3 ± 1.6	19.5 ± 1.6	22.1 ± 2.0	24.8 ± 1.5	<0.001
Severe aortic cusp calcification (Rosenhek IV)	85.1%	80.5%	86.8%	88.1%	0.52
**Distance of annulus to ostium of coronary arteries, mm**					
Left	14.0 ± 3.4	11.7 ± 2.9	14.1 ± 2.6	16.2 ± 3.3	<0.001
Right	17.3 ± 2.9	15.2 ± 2.6	17.6 ± 2.4	19.0 ± 2.6	<0.001
**LVOT, mm**					
Calcification	29%	30%	32%	22%	0.53
Area derived diameter	24.9 ± 2.9	22.1 ± 1.6	24.9 ± 1.8	28.0 ± 1.6	<0.001
Perimeter derived diameter	25.9 ± 3.0	23.2 ± 1.9	25.8 ± 2.0	29.1 ± 1.8	<0.001
Area, mm^2^	496 ± 112	387 ± 54	489 ± 71	620 ± 72	<0.001
Perimeter	81.8 ± 9.3	72.9 ± 5.9	81.1 ± 6.1	91.3 ± 5.8	<0.001
Maximum diameter	29.4 ± 3.4	26.6 ± 2.5	29.2 ± 2.4	32.8 ± 2.5	<0.001
Area sinotubular junction, mm^2^	669 ± 156	559 ± 107	646 ± 111	818 ± 138	<0.001
Area at coronary ostia, mm^2^	808 ± 188	664 ± 107	800 ± 130	967 ± 196	<0.001
Oversizing, mean %	6.7 ± 9.9	4.8 ± 11.3	7.0 ± 10.0	8.1 ± 8.0%	0.20

LVOT = left ventricular outflow tract

### Procedural results and follow-up

The ES3 was successfully implanted in all 235 patients by transfemoral access without general anesthesia and no need for a second valve. There was no procedural death, no coronary obstruction, annular rupture, need intubation or conversion to surgery. Moderate or severe aortic regurgitation after valve implantation was completely absent in all 3 groups assessed by aortography and echocardiography (Table 4). Furthermore, aortic regurgitation index did not differ between groups. Residual mean aortic gradient by echocardiography was significantly lower with 29mm compared with 26mm and 23mm valve sizes translating into a trend towards a higher device success rate according to VARC-2 with the 29mm valve. All 7 cases with device failure were due to a mean aortic gradient >20mmHg. Need for contrast amount was about 90mL with no difference between groups.

**Table 4:** Procedural data.

	All patients	23mm	26mm	29mm	P-Value
Number of patients, N	235	77	91	67	
Nongeneral anesthesia	235 (100%)	77 (100%)	91 (100%)	67 (100%)	—
General anesthesia	0 (0%)	0 (0%)	0 (0%)	0 (0%)	—
Correct placement at intended site	235 (100%)	77 (100%)	91 (100%)	67 (100%)	—
Cardiopulmonary bypass	0 (0%)	0 (0%)	0 (0%)	0 (0%)	—
Aortic regurgitation after valve placement by aortography					
None / trace	219 (93.2%)	73 (94.8%)	81 (89.0%)	65 (97.0%)	0.12
Mild	16 (6.8%)	4 (5.2%)	10 (10.9%)	2 (3.0%)	
Moderate	0	0	0	0	
Severe	0	0	0	0	
Balloon post-dilation	0	0	0	0	—
Implantation of >1 valve	0	0	0	0	—
Adjunctive PCI	0	0	0	0	—
Coronary obstruction	0	0	0	0	—
Annular rupture	0	0	0	0	—
Conversion to surgery	0	0	0	0	—
Aortic regurgitation by echocardiography post TAVR					
None / trace	208 (88.5%)	67 (87.0%)	80 (87.9%)	61 (91.0%)	0.73
Mild	27 (11.5%)	10 (12.9%)	11 (12.1%)	6 (8.9%)	
Moderate	0	0	0	0	
Severe	0	0	0	0	
Mean regurgitation index	24.2 ± 8.9	22.7 ± 8.5	24.7 ± 8.3	25.3 ± 10.0	0.25
Mean aortic gradient, mmHg	12.6 ± 5.0	15.4 ± 5.6	12.0 ± 4.2	10.2 ± 3.6	<0.001
Device success	228 (97.0%)	72 (93.5%)	89 (97.8%)	67 (100%)	0.05
Contras amount, mL	86 ± 24	83 ± 28	91 ± 21	85 ± 23	0.11

Values are mean ± SD or n (%); PCI = percutaneous coronary intervention

For the total population rate of major vascular complications was 3.0% with no need for surgical repair. In 10 patients a covered stent implantation in the common femoral artery was performed to treat failure of the closure system. There was no perforation of the iliac artery. Rate of vascular complications did not differ between the three groups with different sheath sizes. Need for pacemaker implantation due to second (type II) or third degree atrioventricular block was low and did not differ between groups. The total rate for permanent pacemaker implantation was 17.4% including patients with new left bundle branch block plus AV block type I.

Six patients died within 30 days (2 cardiac, 3 non-cardiac, 1 unexplained death). The early safety endpoint at 30 days ranged between 8 to 10% and was similar between groups (Table 5) with no difference in rates of all-cause mortality (2.6% for total population) or disabling and non-disabling stroke (2.1% for total population).

**Table 5:** Thirty days clinical outcome.

	All patients	23mm	26mm	29mm	P-Value
Number of patients, N	235	77	91	67	
Early safety endpoint at 30 days	19 (8.1%)	6 (7.8%)	7 (7.7%)	6 (8.9%)	0.95
All-cause mortality	6 (2.6%)	3 (3.9%)	2 (2.2%)	1 (1.5%)	0.64
Stroke disabling and non-disabling	5 (2.1%)	2 (2.6%)	1 (1.1%)	2 (2.9%)	0.68
Acute kidney injury—stage 2/3	5 (2.1%)	1 (1.3%)	3 (3.3%)	1 (1.5%)	0.59
Major vascular complication	7 (3.0%)	1 (1.3%)	5 (5.5%)	1 (1.5%)	0.22
Valve dysfunction requiring a repeat procedure	0 (0%)	0 (0%)	0 (0%)	0 (0%)	—
Endocarditis	0 (0%)	0 (0%)	0 (0%)	0 (0%)	—
Valve thrombosis	0 (0%)	0 (0%)	0 (0%)	0 (0%)	—
Myocardial infarction	0	0	0	0	—
Implantation of covered stent	10 (4.3%)	2 (2.6%)	6 (6.6%)	2 (2.9%)	0.36
Surgical repair	0	0	0	0	—
Pacemaker implantation*	21 (8.9%)	7 (9.1%)	6 (6.6%)	8 (11.9%)	0.45

Values are mean ± SD or n (%).

* Indication for pacemaker based on second degree (type II) or third degree atrioventricular block

## Discussion

We are able to demonstrate a high rate of device success and low rates of mortality and stroke within 30 days according to VARC-2 criteria for all three ES3 sizes in symptomatic patients undergoing transfemoral TAVI without general anesthesia. With all three valve sizes there was no moderate or severe aortic regurgitation. Mean aortic gradient measured by echocardiography significantly differed and was lowest with the 29mm valve size.

Residual AR after TAVI is a significant independent predictor for long-term mortality [2,3]. With the previous generations of balloon-expandable TAVI prosthesis the risk for moderate or severe AR ranged between 5 to 10% [1,3,16]. Even a mild AR, which occurred in about 38% of patients treated with a balloon-expandable Edwards valve, has been linked to an increased mortality [3]. The new third generation ES3 valve has been designed to eliminate post-procedural AR by an outer skirt [5]. In the very first ES3 experience the rate of moderate or severe AR in 150 patients was 3.8% and rate of mild AR 22.6% [12]. General anesthesia was used in 64% in the 96 patients treated by femoral approach. In addition, surgical cut-down was performed in 4.2% of patients. Based on this early experience, sizing criteria were developed for the 23, 26 and 29mm ES3 valve size [12]. We used these sizing criteria now in our larger patient population of 235 patients treated by transfemoral approach without general anesthesia. We are able to demonstrate that with the use of these sizing criteria [12] in our experienced team the risk of post-procedural moderate or severe AR is eliminated for all 3 valve sizes. With the ES3 no moderate or severe AR was seen in 165 patients [17], 29 [18] patients and 15 patients [19]. We observed mild AR by transthoracic echocardiography one day after TAVI in 11.5% with no statistical difference between the 23, 26 or 29mm valve size. Rate of oversizing according to the annulus area did not differ between groups. Our observation supports the 10.3% rate of mild AR in 165 ES3 patients [17], which is about half the rate of 22.6% observed in the very early experience of 150 patients [12]. An aortic regurgitation index below 25 has been linked to a negative prognostic impact [14]. In our population the post-procedural aortic regurgitation index was below 25 with use of the 23 and 26mm valve but not with the 29mm ES3 valve. Whether this cut-off has the same impact on one year survival for all valve sizes has to be addressed with long-term follow-up. In contrast, Collas et al [20] could not confirm the negative prognostic impact of an AR <25 in a series of 111 patients and Jilaihawi et al [21] showed that in their experience the heart rate adjusted haemodynmic-echocardiographic aortic insufficiency score outperformed the classic AR index.

In addition there was no annular rupture and no conversion to surgery. With a similar STS score the low mortality rate of 2.1% within 30 days observed in the study by Webb et al [12] was confirmed in our larger patient population. There was no difference in all-cause mortality and early safety between the three groups. The occurrence of major vascular bleeding complications was an independently predictor of long-term mortality [4]. To reduce vascular and bleeding complications the diameter of the delivery system was reduced to 14 French for the 23 or 26mm ES3 valve or 16 French for the 29mm ES3 valve. With this improved delivery system we were able to demonstrate a low rate of major vascular complications and no need for surgical vascular repair. Our results confirm the multicenter experience reporting a major vascular complication rate of 4.2% in 96 transfemoral TAVI patients [12]. Vascular complications with the ES3 delivery system are clearly lower compared with the delivery system of previous generations of Edwards prosthesis [4].

The rate of disabling and non-disabling stroke was low with 2.1% in 235 patients and comparable to the rate of 2.7% in the early experience [12]. Numbers are clearly lower compared with the higher stroke rate observed in the PARTNER studies using previous generation devices. The lower stroke rate may be also linked to lower enhanced platelet activation with the ES3 compared with the Edwards XT possibly triggered by a lower rate of AR [22]. Whether the elimination of post-procedural moderate and severe AR, the reduction of post-procedural mild AR, the lower rate of major vascular complications and the lower rate of stroke do have the impact to reduce long-term mortality with the ES3 compared with previous generations has to be answered by long-term follow-ups.

### Conclusion

In patients with severe aortic stenosis transfemoral TAVI with the ES3 valve without general anesthesia was associated with a high rate of device success, no moderate or severe residual aortic regurgitation, low rates of major vascular complication, mortality and stroke within 30 days with no difference between the 3 valve sizes.

## Supporting Information

S1 CONSORT Checklist CONSORT Checklist. (PDF)

S1 Protocol Trial Protocol. (PDF)

S2 Protocol Trial Protocol in German. (PDF)
