# Supplementary material for: Transfemoral Aortic Valve Implantation with the New Edwards Sapien 3 Valve for Treatment of Severe Aortic Stenosis—Impact of Valve Size in a Single Center Experience
Source: PLoS One. 2016 Mar 22;11(3):e0151247. doi: 10.1371/journal.pone.0151247 (PMC4803290; doi:10.1371/journal.pone.0151247)
Supplement: S1 Protocol — (PDF) [file pone.0151247.s002.pdf]

CSI-Ulm TAVI

Clinicaltrials.gov NCT02162069

1. Title

Coronary and structural interventions Ulm – transcatheter aortic valve implantation

Principal investigator: Prof. Dr. med. Jochen Wöhrle
Head Interventional Cardiology Research Group
Director Structural Heart Valve Program
Director Catheterization Laboratories
Dept. of Internal Medicine II
University of Ulm, Ulm, Germany

This is a prospectiv and single center study.

2. Protocol summary

Aim of this study is the evaluation of acute and long-term outcomes after transcatheter aortic valve implantation at the University of Ulm, Ulm, Germany.

All outcome data will be evaluated according to the VARC-2 criteria (J Am Coll Cardiol 2012;60:1438–54):

**Table 2**	**Mortality**
**All-cause mortality**	
**Cardiovascular mortality**	
Any of the following criteria:	
Death due to proximate cardiac cause (e.g. myocardial infarction, cardiac tamponade, worsening heart failure)	
Death caused by non-coronary vascular conditions such as neurological events, pulmonary embolism, ruptured aortic aneurysm, dissecting aneurysm, or other vascular disease	
All procedure-related deaths, including those related to a complication of the procedure or treatment for a complication of the procedure	
All valve-related deaths including structural or non-structural valve dysfunction or other valve-related adverse events	
Sudden or unwitnessed death	
Death of unknown cause	
**Non-cardiovascular mortality**	
Any death in which the primary cause of death is clearly related to another condition (e.g. trauma, cancer, suicide)	

Table 3 Myocardial Infarction

Peri-procedural MI (<72 h after the index procedure)

- New ischemic symptoms (e.g. chest pain or shortness of breath), or new ischemic signs (e.g. ventricular arrhythmias, new or worsening heart failure, new ST-segment changes, hemodynamic instability, new pathological Q-waves in at least two contiguous leads, imaging evidence of new loss of viable myocardium or new wall motion abnormality) AND
- Elevated cardiac biomarkers (preferable CK-MB) within 72 h after the index procedure, consisting of at least one sample post-procedure with a peak value exceeding 15 x as the upper reference limit for troponin or 5 x for CK-MB.* If cardiac biomarkers are increased at baseline (>99th percentile), a further increase in at least 50% post-procedure is required AND the peak value must exceed the previously stated limit

Spontaneous MI (>72 h after the index procedure)

- Any one of the following criteria
- Detection of rise and/or fall of cardiac biomarkers (preferably troponin) with at least one value above the 99th percentile URL, together with the evidence of myocardial ischemia with at least one of the following:
  - Symptoms of ischemia
  - ECG changes indicative of new ischemia [new ST-T changes or new left bundle branch block (LBBB)]
  - New pathological Q-waves in at least two contiguous leads
  - Imaging evidence of a new loss of viable myocardium or new wall motion abnormality
- Sudden, unexpected cardiac death, involving cardiac arrest, often with symptoms suggestive of myocardial ischemia, and accompanied by presumably new ST elevation, or new LBBB, and/ or evidence of fresh thrombus by coronary angiography and/or at autopsy, but death occurring before blood samples could be obtained, or at a time before the appearance of cardiac biomarkers in the blood.
- Pathological findings of an acute myocardial infarction

Table 4 Stroke and TIA

Diagnostic criteria

- Acute episode of a focal or global neurological deficit with at least one of the following: change in the level of consciousness, hemiplegia, hemiparesis, numbness, or sensory loss affecting one side of the body, dysphasia or aphasia, hemianopia, amaurosis fugax, or other neurological signs or symptoms consistent with stroke
- Stroke: duration of a focal or global neurological deficit >24 h; OR <24 h if available neuroimaging documents a new haemorrhage or infarct; OR the neurological deficit results in death
- TIA: duration of a focal or global neurological deficit <24 h, any variable neuroimaging does not demonstrate a new hemorrhage or infarct
- No other readily identifiable non-stroke cause for the clinical presentation (e.g. brain tumour, trauma, infection, hypoglycemia, peripheral lesion, pharmacological influences), to be determined by or in conjunction with the designated neurologist*
- Confirmation of the diagnosis by at least one of the following:
  - Neurologist or neurosurgical specialist
  - Neuroimaging procedure (CT scan or brain MRI), but stroke may be diagnosed on clinical grounds alone

Stroke classification

- Ischemic: an acute episode of focal cerebral, spinal, or retinal dysfunction caused by infarction of the central nervous system tissue
- Hemorrhagic: an acute episode of focal or global cerebral or spinal dysfunction caused by intraparenchymal, intraventricular, or subarachnoid hemorrhage
- A stroke may be classified as undetermined if there is insufficient information to allow categorization as ischemic or haemorrhagic

Stroke definitions[†]

- Disabling stroke: an mRS score of 2 or more at 90 days and an increase in at least one mRS category from an individual's pre-stroke baseline
- Non-disabling stroke: an mRS score of <2 at 90 days or one that does not result in an increase in at least one mRS category from an individual's pre-stroke baseline

Table 11	Composite Endpoints
**Device success**	
Absence of procedural mortality AND	
Correct positioning of a single prosthetic heart valve into the proper anatomical location AND	
Intended performance of the prosthetic heart valve (no prosthesis- patient mismatch* and mean aortic valve gradient <20 mmHg or peak velocity <3 m/s, AND no moderate or severe prosthetic valve regurgitation*)	
**Early safety (at 30 days)**	
All-cause mortality	
All stroke (disabling and non-disabling)	
Life-threatening bleeding	
Acute kidney injury—Stage 2 or 3 (including renal replacement therapy)	
Coronary artery obstruction requiring intervention	
Major vascular complication	
Valve-related dysfunction requiring repeat procedure (BAV, TAVI, or SAVR)	
**Clinical efficacy (after 30 days)**	
All-cause mortality	
All stroke (disabling and non-disabling)	
Requiring hospitalizations for valve-related symptoms or worsening congestive heart failure‡	
NYHA class III or IV	
Valve-related dysfunction (mean aortic valve gradient >20 mmHg, EOA <0.9-1.1 cm^2† and/or DVI <0. 35 m/s, AND/OR moderate or severe prosthetic valve regurgitation*)	
**Time-related valve safety**	
Structural valve deterioration	
Valve-related dysfunction (mean aortic valve gradient >20 mm Hg, EOA <0.9–1.1 cm^2‡ and/or DVI <0.35 m/s, AND/ OR moderate or severe prosthetic valve regurgitation*)	
Requiring repeat procedure (TAVI or SAVR)	
Prosthetic valve endocarditis	
Prosthetic valve thrombosis	
Thrombo-embolic events (e.g. stroke)	
VARC bleeding, unless clearly unrelated to valve therapy (e.g. trauma)	

Literatur:

1. Iung B, Baron G, Butchart EG, Delahaye F, Gohlke-Bärwolf C, Levang OW, Tornos P, Vanoverschelde JL, Vermeer F, Boersma E, Ravaud P, Vahanian A. A prospective survey of patients with valvular heart disease in Europe: the Euro Heart Survey on Valvular Heart Disease. Eur Heart J 2003;24:1231–1243.

2. Nkomo VT, Gardin JM, Skelton TN, Gottdiener JS, Scott CG, Enriquez-Sarano M. Burden of valvular heart diseases: a population-based study. Lancet 2006;368:1005–1011.

3. Rosenhek R, Binder T, Porenta G, Lang I, Christ G, Schemper M, Maurer G, Baumgartner H. Predictors of outcome in severe, asymptomatic aortic stenosis.
N Engl J Med 2000;343:611 – 617.

4. Pellikka PA, Sarano ME, Nishimura RA, Malouf JF, Bailey KR, Scott CG, Barnes ME, Tajik AJ. Outcome of 622 adults with asymptomatic, hemodynamically significant aortic stenosis during prolonged follow-up. Circulation 2005;111: 3290 – 3295.

5. Horstkotte D, Loogen F. The natural history of aortic valve stenosis. Eur Heart J 1988;9 Suppl E:57– 64.

6. 14. Bouma BJ, van den Brink RBA, van der Meulen JHP, et al. To operate or not on elderly patients with aortic stenosis: the decision and its consequences. Heart 1999; 82:143-8.

7. Iung B, Cachier A, Baron G, et al. Decision-making in elderly patients with severe aortic stenosis: why are so many denied surgery? Eur Heart J 2005;26:2714- 20.

8. Kappetein AP, Head SJ, Généreux P, et al. Updated standardized endpoint definitions for transcatheter aortic valve implantation: the Valve Academic Research Consortium-2 consensus document. J Am Coll Cardiol. 2012 Oct 9;60(15):1438-54
