# Supplementary material for: Transfemoral Aortic Valve Implantation with the New Edwards Sapien 3 Valve for Treatment of Severe Aortic Stenosis—Impact of Valve Size in a Single Center Experience
Source: PLoS One. 2016 Mar 22;11(3):e0151247. doi: 10.1371/journal.pone.0151247 (PMC4803290; doi:10.1371/journal.pone.0151247)
Supplement: S2 Protocol — (PDF) [file pone.0151247.s003.pdf]

Darstellung von Forschungsprojekten über wissenschaftliche Untersuchungen am Menschen

1. Titel des Projektes:

Langzeitresultate nach perkutanem Herzklappenersatz am Universitätsklinikum Ulm

Studienleitung: Prof. Dr. med. Jochen Wöhrle
Leitender Oberarzt
Klinik für Innere Medizin II

Es handelt sich um eine prospektives monozentrisches Forschungsprojekt.

2. Beschreibung des Forschungsprogrammes:

Die hochgradige Aortenklappenstenose ist der häufigste Herzklappenfehler in der westlichen Welt. Sie tritt am häufigsten als erworbene kalzifizierte Aortenklappenstenose bei älteren Erwachsenen auf. Die Prävalenz liegt bei 2,7% der Bevölkerung über 65 Jahre (1,2). Die erworbene Aortenklappenstenose ist eine chronische, progressive Erkrankung, die über einen langen Zeitraum hinweg asymptomatisch bleiben kann. Es kommt durch eine zunehmende Verkalkung der Aortenklappentaschen zu einer Verkleinerung der Aortenklappenöffnungsfläche, (die physiologischerweise 3-4 cm^2 beträgt) auf eine Fläche kleiner 1,0cm^2. Der linke Ventrikel muss daher mehr Druck aufbauen, um das Blut durch die Aortenklappe in die Aorta zu pumpen, was zunächst in der Regel zu einer Hypertrophie des linken Ventrikels führt. Zu diesem Zeitpunkt sind die Patienten meist noch asymptomatisch. Ist der linke Ventrikel dieser Mehrarbeit aber nicht mehr gewachsen, kommt es zum Auftreten von Symptomen wie Zeichen der Herzinsuffizienz, Auftreten von Angina pectoris, Schwindel oder Synkopen. Schon bei einer asymptomatischen hochgradigen Aortenklappenstenose besteht eine eingeschränkte Lebenserwartung, in der Regel bedingt durch das Auftreten des plötzlichen Herztodes. Die Angabe der Überlebensrate variiert in der Literatur zwischen 20-50% nach 5 Jahren (3,4). Sobald die Erkrankung aber symptomatisch wird, nimmt die Prognose deutlich ab, es besteht nur noch eine mittlere Überlebenszeit von 2 Jahren, nach 5 Jahren liegt die Überlebenswahrscheinlichkeit bei nur noch 18 +/- 7%. (5).
Eine konservative Therapieoption besteht nicht. Vor Einführung der perkutanen Aortenklappenimplantation (TAVI) vor 12 Jahren (seit 2007 am Universitätsklinikum Ulm) in die klinische Routineversorgung konnten Patienten mit einer hochgradigen Aortenklappenstenose ausschließlich durch eine Operation am offenen Herzen unter Einsatz einer Herz-LungenMaschine und Ersatz der Herzklappe behandelt werden. Aufgrund des höheren Alters der Patienten und der bestehenden Komorbiditäten geht dies jedoch häufig mit einem deutlich erhöhten perioperativen Risiko einher, so daß die

konventionelle Operation in mindestens 30% der Patienten keine Therapieoption darstellte (6,7).

Bezüglich des perkutanen Aortenklappenersatzes liegen fundierte randomisierte Studie vor, welche diese Therapie in den letzten Jahren in die klinische Routineversorgung in Deutschland transportiert haben. Ziel dieses Forschungsprojektes ist es die Akut- und Langzeitdaten nach perkutanem Herzklappenersatz am Universitätsklinikum Ulm zu erfassen anhand der VARC-2 Kriterien.

In der klinischen Routine wird vor einem perkutanen Aortenklappenersatz eine EKG, Belastungs-EKG, Echokardiographie, Rechts-Links-Herzkatheter, Herz-CT und ggf. Herz-MRT durchgeführt. Diese standardisierte Bildgebung dient der genauen Vermessung der Aortenklappenstenose (v.a. im Bereich des sogenannten Anulus = Ende der Aortenklappensegel im linksventrikulären Ausflusstrakt), um eine entsprechenden Typ und Größe einer perkutanen Herzklappe auswählen zu können. Für die Behandlung von Patienten mit einer perkutanen Herzklappe werden aktuell vor allem 3 verschieden Typen von Herzklappen (Lotus, Edwards Sapien 3, Corevalve) in allen verfügbaren Größen vorgehalten. Die Auswahl erfolgt nach Vermessung des Anulus und des Zugangsweges (transfemoral rechts / links, transapikal) entsprechend der klinischen Standards um ein optimales Resultat zu erzielen.

Nach dem perkutanen Aortenklappenersatz wird in den ersten drei Tagen jeweils ein EKG und eine Echokardiographie durchgeführt.

Alle Patienten mit einem Herzklappenersatz aus der Region Ulm werden in der kardiologischen Ambulanz nach 4-6 Wochen, 3, 6, 12 Monaten und nachfolgend jährlich weiter betreut. Hierbei wird neben dem EKG, Belastungs-EKG jeweils eine Echokardiographie durchgeführt. Nach klinischer Indikation bei weiteren Fragestellungen ggf. ein Kardio-MRT / Herz-CT z.B. bei der Frage ob vorhanden Koronarstenosen zu einer nachweisbaren Ischämie führen und behandelt werden müssen.

Ziel des beantragten Forschungsprojektes ist es alle Patienten vor einem Herzklappenersatz zu fragen, ob diese erhobenen Daten dokumentiert und analysiert werden dürfen.

Wissenschaftliches Ziel des Forschungsprojektes ist es:

1. Die Resultate der perkutanen Herzklappentherapie am Universitätsklinikum Ulm prospektiv zu erfassen und mit publizierten Daten zu vergleichen. Hierbei werden die Ereignisse der sogenannten VARC Kriterien erfasst (8). Bei den VARC Kriterien handelt es sich um von einem internationalen Academic Research Consortium erstellten Kriterien, bei welchen der Erfolg des Eingriffes als auch unerwünschte Ereignisse definiert sind. Durch die Erfassung der Ereignisse auf Basis der definierten VARC Kriterien können die Resultate verschiedener Forschungsgruppen und Publikationen sinnvoll miteinander verglichen werden, da z.B. das Auftreten eines Schlaganfalles oder eines Herzinfarktes der gleichen Definition unterliegen. Die Publikation der VARC Kriterien ist diesem Antrag beigefügt.

2. Durch die Überprüfung der Resultate wichtige Kriterien zu identifizieren, welche zukünftig die perkutane Herzklappentherapie am Universitätsklinikum Ulm weiter verbessern.
    a. Evaluation der sogenannten paravalvulären Insuffizienz bestimmt durch die Echokardiographie
    b. Häufigkeit des Auftretens von Vorhofflimmern, Linksschenkelblock und AV-Blockierungen (dokumentiert im EKG)
    c. Evaluation von ggf. vorhandenen Unterschieden zwischen den verwendeten Herzklappen

Nachfolgend sind alle VARC Ereignisse (und Definitionen) aus J Am Coll Cardiol 2012; 60:1438–54 dargestellt, welche im Rahmen dieses Forschungsprojektes prospektiv erfasst werden sollen (8).

Table 2	Mortality
All-cause mortality	
Cardiovascular mortality	
Any of the following criteria:	
Death due to proximate cardiac cause (e.g. myocardial infarction, cardiac tamponade, worsening heart failure)	
Death caused by non-coronary vascular conditions such as neurological events, pulmonary embolism, ruptured aortic aneurysm, dissecting aneurysm, or other vascular disease	
All procedure-related deaths, including those related to a complication of the procedure or treatment for a complication of the procedure	
All valve-related deaths including structural or non-structural valve dysfunction or other valve-related adverse events	
Sudden or unwitnessed death	
Death of unknown cause	
Non-cardiovascular mortality	
Any death in which the primary cause of death is clearly related to another condition (e.g. trauma, cancer, suicide)	

Table 3 Myocardial Infarction

Peri-procedural MI (<72 h after the index procedure)

- New ischemic symptoms (e.g. chest pain or shortness of breath), or new ischemic signs (e.g. ventricular arrhythmias, new or worsening heart failure, new ST-segment changes, hemodynamic instability, new pathological Q-waves in at least two contiguous leads, imaging evidence of new loss of viable myocardium or new wall motion abnormality) AND
- Elevated cardiac biomarkers (preferable CK-MB) within 72 h after the index procedure, consisting of at least one sample post-procedure with a peak value exceeding 15 x as the upper reference limit for troponin or 5 x for CK-MB.* If cardiac biomarkers are increased at baseline (>99th percentile), a further increase in at least 50% post-procedure is required AND the peak value must exceed the previously stated limit

Spontaneous MI (>72 h after the index procedure)

- Any one of the following criteria
- Detection of rise and/or fall of cardiac biomarkers (preferably troponin) with at least one value above the 99th percentile URL, together with the evidence of myocardial ischemia with at least one of the following:
  - Symptoms of ischemia
  - ECG changes indicative of new ischemia [new ST-T changes or new left bundle branch block (LBBB)]
  - New pathological Q-waves in at least two contiguous leads
  - Imaging evidence of a new loss of viable myocardium or new wall motion abnormality
- Sudden, unexpected cardiac death, involving cardiac arrest, often with symptoms suggestive of myocardial ischemia, and accompanied by presumably new ST elevation, or new LBBB, and/ or evidence of fresh thrombus by coronary angiography and/or at autopsy, but death occurring before blood samples could be obtained, or at a time before the appearance of cardiac biomarkers in the blood.
- Pathological findings of an acute myocardial infarction

Table 4	Stroke and TIA

Diagnostic criteria

- Acute episode of a focal or global neurological deficit with at least one of the following: change in the level of consciousness, hemiplegia, hemiparesis, numbness, or sensory loss affecting one side of the body, dysphasia or aphasia, hemianopia, amaurosis fugax, or other neurological signs or symptoms consistent with stroke
- Stroke: duration of a focal or global neurological deficit >24 h; OR <24 h if available neuroimaging documents a new haemorrhage or infarct; OR the neurological deficit results in death
- TIA: duration of a focal or global neurological deficit <24 h, any variable neuroimaging does not demonstrate a new hemorrhage or infarct
- No other readily identifiable non-stroke cause for the clinical presentation (e.g. brain tumour, trauma, infection, hypoglycemia, peripheral lesion, pharmacological influences), to be determined by or in conjunction with the designated neurologist*
- Confirmation of the diagnosis by at least one of the following:
  - Neurologist or neurosurgical specialist
  - Neuroimaging procedure (CT scan or brain MRI), but stroke may be diagnosed on clinical grounds alone

Stroke classification

- Ischemic: an acute episode of focal cerebral, spinal, or retinal dysfunction caused by infarction of the central nervous system tissue
- Hemorrhagic: an acute episode of focal or global cerebral or spinal dysfunction caused by intraparenchymal, intraventricular, or subarachnoid hemorrhage
- A stroke may be classified as undetermined if there is insufficient information to allow categorization as ischemic or haemorrhagic

Stroke definitions†

- Disabling stroke: an mRS score of 2 or more at 90 days and an increase in at least one mRS category from an individual's pre-stroke baseline
- Non-disabling stroke: an mRS score of <2 at 90 days or one that does not result in an increase in at least one mRS category from an individual's pre-stroke baseline

Table 11 Composite Endpoints
**Device success**
Absence of procedural mortality AND
Correct positioning of a single prosthetic heart valve into the proper anatomical location AND
Intended performance of the prosthetic heart valve (no prosthesis- patient mismatch* and mean aortic valve gradient <20 mmHg or peak velocity <3 m/s, AND no moderate or severe prosthetic valve regurgitation*)
**Early safety (at 30 days)**
All-cause mortality
All stroke (disabling and non-disabling)
Life-threatening bleeding
Acute kidney injury—Stage 2 or 3 (including renal replacement therapy)
Coronary artery obstruction requiring intervention
Major vascular complication
Valve-related dysfunction requiring repeat procedure (BAV, TAVI, or SAVR)
**Clinical efficacy (after 30 days)**
All-cause mortality
All stroke (disabling and non-disabling)
Requiring hospitalizations for valve-related symptoms or worsening congestive heart failure‡
NYHA class III or IV
Valve-related dysfunction (mean aortic valve gradient >20 mmHg, EOA <0.9-1.1 cm^{2}† and/or DVI <0. 35 m/s, AND/OR moderate or severe prosthetic valve regurgitation*)
**Time-related valve safety**
Structural valve deterioration
Valve-related dysfunction (mean aortic valve gradient >20 mm Hg, EOA <0.9–1.1 cm^{2}‡ and/or DVI <0.35 m/s, AND/ OR moderate or severe prosthetic valve regurgitation*)
Requiring repeat procedure (TAVI or SAVR)
Prosthetic valve endocarditis
Prosthetic valve thrombosis
Thrombo-embolic events (e.g. stroke)
VARC bleeding, unless clearly unrelated to valve therapy (e.g. trauma)

<u>Literatur:</u>

1. Iung B, Baron G, Butchart EG, Delahaye F, Gohlke-Bärwolf C, Levang OW, Tornos P, Vanoverschelde JL, Vermeer F, Boersma E, Ravaud P, Vahanian A. A prospective survey of patients with valvular heart disease in Europe: the Euro Heart Survey on Valvular Heart Disease. Eur Heart J 2003;24:1231–1243.

2. Nkomo VT, Gardin JM, Skelton TN, Gottdiener JS, Scott CG, Enriquez-Sarano M. Burden of valvular heart diseases: a population-based study. Lancet 2006;368:1005–1011.

3. Rosenhek R, Binder T, Porenta G, Lang I, Christ G, Schemper M, Maurer G, Baumgartner H. Predictors of outcome in severe, asymptomatic aortic stenosis.
N Engl J Med 2000;343:611 – 617.

4. Pellikka PA, Sarano ME, Nishimura RA, Malouf JF, Bailey KR, Scott CG, Barnes ME, Tajik AJ. Outcome of 622 adults with asymptomatic, hemodynamically significant aortic stenosis during prolonged follow-up. Circulation 2005;111: 3290 – 3295.

5. Horstkotte D, Loogen F. The natural history of aortic valve stenosis. Eur Heart J 1988;9 Suppl E:57– 64.

6. 14. Bouma BJ, van den Brink RBA, van der Meulen JHP, et al. To operate or not on elderly patients with aortic stenosis: the decision and its consequences. Heart 1999; 82:143-8.

7. Iung B, Cachier A, Baron G, et al. Decision-making in elderly patients with severe aortic stenosis: why are so many denied surgery? Eur Heart J 2005;26:2714- 20.

8. Kappetein AP, Head SJ, Généreux P, et al. Updated standardized endpoint definitions for transcatheter aortic valve implantation: the Valve Academic Research Consortium-2 consensus document. J Am Coll Cardiol. 2012 Oct 9;60(15):1438-54
